# Common conditions affecting the ocular surface

**Published:** 2024-10-02

**Authors:** Victor Hu

**Affiliations:** 1 International Centre for Eye Health, London School of Hygiene and Tropical Medicine UK.

**Improving our understanding and management of ocular surface conditions, and educating patients about what they can do, will enhance patients’ quality of life and protect their sight.**

**Figure:**
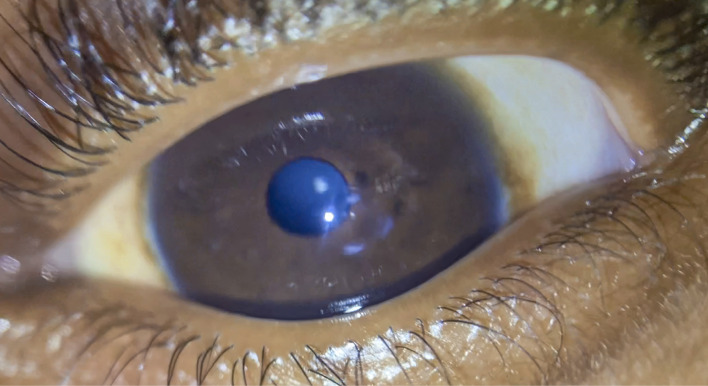
Keratoconus, noticeable as circular scarring near the centre of the cornea. The pigmented limbus suggests chronic allergic eye disease, a major risk factor for the development of keratoconus.

© ANDREW BLAIKIE CC BY-NC-SA 4.0

The ocular surface consists of the ocular structures which are directly in contact with the outside world, including the cornea, conjunctiva, eyelids and eyelashes, the tear film, and any associated glands. A healthy ocular surface is vital for both comfort and clear vision.

Many patients with conditions affecting the ocular surface – such as dry eye and allergic conjunctivitis – do not experience significant sight loss. However, they often make up the greatest number of patients seen by eye care services. Even in relatively mild forms, these conditions can cause significant discomfort and affect patients’ quality of life. If left untreated, they can cause changes to the corneal surface that may permanently impair vision in one or both eyes.

Dry eye disease is estimated to affect between 5% and 50% of people, often resulting in constant, debilitating irritation for those affected.^1^ Children and some adults commonly experience ocular allergies, which can cause intense itching and inflammation. These symptoms can be distressing, leading to social withdrawal and children missing school. Ocular allergy and eye rubbing are also strongly associated with keratoconus, a condition in which progressive thinning of the cornea occurs, potentially resulting in loss of vision.

This issue offers practical, clear guidance on managing dry eye, allergic eye disease, and keratoconus, including recent advances such as corneal cross-linking.

*Image couresy of Andrew Blaikie, University of St Andrews.*
